# Impacts of the Accreditation Process for Undergraduate Medical Schools: A Scoping Review

**DOI:** 10.1111/tct.70031

**Published:** 2025-02-13

**Authors:** Leticia C. Girotto, Karynne B. Machado, Roberta F. C. Moreira, Milton A. Martins, Patrícia Z. Tempski

**Affiliations:** ^1^ Center for Development of Medical Education University of São Paulo São Paulo Brazil; ^2^ University of São Paulo São Paulo Brazil; ^3^ Hospital Sirio Libanês São Paulo Brazil; ^4^ Department of Medicine University of São Paulo São Paulo Brazil

**Keywords:** accreditation, evaluation, undergraduate medical education

## Abstract

**Objective:**

The objective of this study is to identify, synthesize and communicate research findings; identify research gaps; and formulate recommendations for future research regarding the impact of the accreditation process on undergraduate medical schools around the world.

**Method:**

This scoping review followed the recommendations of the Preferred Reporting Items for Systematic Reviews and Meta‐Analyses–extension for scoping reviews (PRISMA‐ScR). The electronic search was performed up to March 2024 to identify studies that investigated the impact of the accreditation process on undergraduate medical schools. Two independent reviewers performed the selection process and extracted data from the included studies to perform a qualitative analysis.

**Results:**

The search identified 5148 references. After the selection process, 31 studies from 14 countries were included for data extraction. Studies highlighted five main themes: curricular and governance changes, continuous quality improvement, students' performance, school recognition and student satisfaction, and accreditation data sharing proposal. Three studies presented negative points related to accreditation process.

**Conclusion:**

The accreditation process induced both positive impacts and negative burdens for undergraduate medical schools. Continuous quality improvement is a common element identified by studies to help stakeholders ensure that accreditation improves the quality of medical education and, consequently, the health care provided. Experiences with accreditation should be shared and reported to improve the quality of medical education worldwide. Future studies must be carried out with a clear description of the domains and criteria considered during the accreditation process as well as the outcome measures used.

## 1 Introduction

The quality of medical education is on the agenda of education and health authorities worldwide, and challenges with maintaining high‐quality education have become even more evident with the increasing number of medical schools in recent years. The World Directory of Medical Schools, an international database developed by the Foundation for the Advancement of International Medical Education and Research (FAIMER) and the World Federation for Medical Education (WFME), listed 3602 medical schools around the world through 1 June 2016 (https://www.wdoms.org/).

Considering this scenario, it is necessary to establish minimum standards that must be incorporated into all medical school curricula, focusing on ensuring the highest possible quality of professional training [1] and, consequently, of the health care provided [2, 3].

Focusing on this issue, the WFME, with support from the World Health Organization (WHO), recommends accreditation as an effective tool for quality assurance [4]. Accreditation is a process by which a medical school is evaluated using a set of clear criteria to ensure the quality of medical education and promote improvements, with the goal of providing optimal patient care [5, 6].

There has been a substantial increase in the accreditation of medical schools in many countries in recent years. However, a considerable number of medical schools listed in the World Directory of Medical Schools do not have accreditation systems for medical education. The reasons include an unawareness of the advantages of accreditation, insufficient resources, a lack of incentives, and other political factors [7].

The accreditation of medical education has been considered to result in competent practising doctors.

However, few data show evidence that the accreditation of medical schools has a positive impact on the quality of medical education. More important than episodic accreditation, the development of a process of continuous quality improvement of medical schools can positively influence the quality of medical education worldwide.

Continuous Quality Improvement is presented by the evaluation instrument of the Liaison Committee on Medical Education (LCME), the accreditation system for medical schools in the USA and Canada as ‘A medical school engages in continuous planning and continuous quality improvement processes that establish programmatic goals short‐ and long‐term, result in the achievement of measurable results that are used to improve programmatic quality and ensure effective monitoring of medical education program compliance with accreditation standards’ [8].

The definition presented for CQI brings the concept of impact, that is, permanent and measurable transformations directly related to an experienced process [9]. Results, in turn, can be transitory and not necessarily promote impacts. Regarding the accreditation process, what is expected is that it promotes permanent transformations and generates positive impacts on medical education [10].

Thus, this scoping review aimed to identify, synthesize and communicate the research findings about the impact of the accreditation process on undergraduate medical schools around the world. In addition, we aimed to identify research gaps and to formulate recommendations for future research. For this purpose, we focused on the main question: (1) What is the evidence pertaining to the impact of the accreditation process on medical schools worldwide?

## 2 Method

### 2.1 Design and Setting

We performed a scoping review of the literature in accordance with the Cochrane Collaboration Guidelines [11] and following the recommendations for conducting scoping reviews [12, 13]. This scoping review was reported following the recommendations of the Preferred Reporting Items for Systematic Reviews and Meta‐Analyses–extension for scoping reviews (PRISMA‐ScR) [12].

Scoping reviews are used to investigate emerging issues and report on the type and volume of evidence available in the literature, in addition to identifying key points, clarifying concepts and presenting knowledge gaps [13].

### 2.2 Criteria for Inclusion of Studies

The question of interest for this review was structured using the acronym PCC, which guided the eligibility criteria as follows:

- P (population, condition): stakeholders involved in undergraduate medical education. Thus, studies that did not include medical students or medical schools at the undergraduate level were excluded.
- C (concept): identification of the consequences or effects (impacts) of the accreditation process.
- C (context): medical school accreditation process at the undergraduate level.

Thus, any primary (descriptive or analytical) or secondary study that assessed stakeholders' perceptions about the impacts of the accreditation process for medical schools was considered. All studies published in the databases before March 2024 were included, and there was no restriction regarding the date of publication or language.

### 2.3 Searching for Studies

An electronic search of electronic databases was performed in the PubMed, EMBASE, CINAHL, Web of Science, Scopus, Lilacs, ERIC, Cochrane Library, World Health Organization's Institutional Repository for Information Sharing (WHO IRIS), Centro Nacional de Informação de Ciências Médicas de Cuba (CUMED), COLECIONASUS, Índice Bibliográfico Español en Ciencias de la Salud (IBECS) Bibliografia Nacional em Ciencias de Ia Salud Argentina (BINACIS) e Índice Bibliográfico Español en Ciencias de la Salud (IBECS) and Grey Literature and Open Grey databases using the following keyword combinations: (Medical Schools OR Medical School OR School, Medical) AND (accreditation) AND (Improvement, Quality OR Improvements, Quality OR Quality Improvements). To select the terms, we consulted the controlled vocabulary thesaurus Medical Subject Headings (MeSH). The searches were completed in October 2021, and an update was performed in March 2024. A detailed appendix with search strategy is shown in Table A1.

The search for studies followed the three‐step method recommended by the Joanna Briggs Institute [14]. Thus, references retrieved through the initial electronic search have their titles and abstracts analysed to identify other appropriate keywords to follow the electronic search in all selected databases. Finally, the third step was taken through the search on the reference list of all included studies [14].

### 2.4 Selecting Studies

The study selection process was carried out in two phases using the Rayyan platform. The first phase consisted of reading the titles and abstracts of all references retrieved by the search strategies and categorizing the studies as ‘potentially eligible’ or ‘eliminated’. The second phase consisted of reading in full the ‘potentially eligible’ studies to confirm their eligibility or exclude them in the second phase (the justifications for each exclusion in the second phase are presented). The two phases were conducted by two independent researchers (LCG and KBM), and inconsistencies in decisions to include or exclude studies were resolved by a third researcher (PZT). The entire selection process is presented using a PRISMA flowchart. We independently analysed the studies that met the inclusion criteria and checked their reference lists to identify potentially relevant studies that were not retrieved from the electronic search.

### 2.5 Extracting Data

We developed a standardized form to record the characteristics of the included studies and the key information relevant to the review question. The following information was collected: (1) author(s), (2) year of publication, (3) country of origin, (4) aims/purpose, (5) type of study, (6) methodology used to measure outcomes and (7) key findings related to the review question.

### 2.6 Quality Assessment/Risk of Bias of the Included Studies

As the aim of this scoping review is to map the impacts of undergraduate medical school accreditation reported by descriptive studies or to use pieces of analytical studies reporting the impacts of undergraduate medical school accreditation, no checklists or tools for assessing the methodological quality of the studies were applied [15].

### 2.7 Synthesis and Presentation of Results

The results (extracted data) are presented in table format. According to the results presented by the studies, they were categorized and discussed in the form of a narrative synthesis.

## 3 Results

### 3.1 Search Results

**FIGURE 1:**
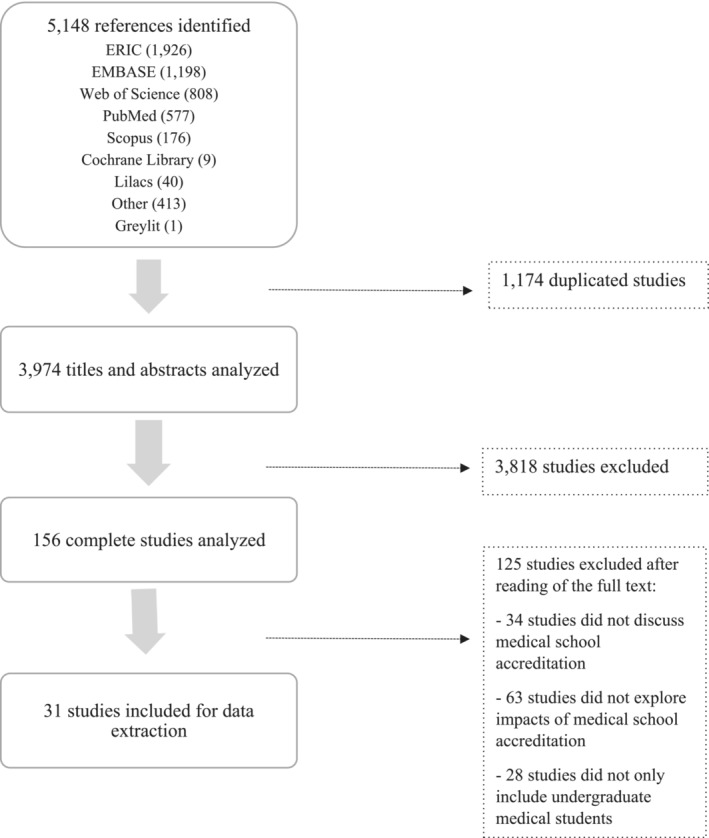
Flowchart of the study selection process.

Our electronic search yielded 5148 references. At the end of the selection process, we included 31 studies (Figure 1). The extraction form shown in Table 1 presents detailed information about the author, year of publication, country of origin, study aim or purpose, type of study, methodology adopted to measure outcomes, key findings and categorization regarding the impacts. The main characteristics of the included studies/documents are detailed in Table 1.

**TABLE 1:** Extracted data of the selected studies.

*n*	Author(s)	Year	Country of origin	Objective/purpose	Type of study	Methodology for measuring results	Main conclusions	Categorization regarding the impacts of accreditation
1	Alrebish SA, Jolly bc, Molloy EK	2017	Saudi Arabia	To analyse the proposals and results of accreditation in 6 medical schools in Saudi Arabia and the views of stakeholders on the process	Qualitative, with focus groups and interviews	Data triangulation:bibliographic review, document analysis, focus groups with students and teachers and interview with coordinator	Negative points: stress, temporary changes to comply with the checklist, accreditation agency not being specific to medicine (it is for all undergraduate courses); Positive points: assessment culture, ‘sustainable accreditation’.	CQI Negative points
2	Barzansky B, Hunt D, Moineau G, et al.	2015	United States	Identify factors that affect the implementation of CQI processes during an accreditation process and other	Observational and descriptive—case series	Review of case reports	Accreditation fosters a culture of CQI, which in turn strengthens the quality of the educational programme and its outcomes.	CQI
3	Blouin D, Tekian A, Kamin C, Harris IB.	2018	Canada	Assess the impact of accreditation, identifying the processes implemented by the school as a result of accreditation	Qualitative with focus groups and interviews	Interviews and focus groups	Strengths: governance, academic accountability, data collection and analysis, monitoring, documentation and policy review creation, CQI, and faculty engagement Negative points: demand for financial and human resources that leads to loss of investment opportunities in other areas, potential impact on faculty and staff morale, dissatisfaction with the current accreditation process.	CQI Negative points Curriculum and governance changes
4	Blouin D.	2020	Canada	Identify factors highlighted by the actors involved that indicate the effectiveness of accreditation	Qualitative with focus groups and interviews	Interviews and focus groups	Direct interference: process change, CQI and programme quality, research. Indirect interference: stakeholder satisfaction, student and alumni performance, stakeholder engagement and expectations, research.	CQI Curriculum and governance changes Student performance School recognition/Student satisfaction
5	Chandran L, Fleit HB, Shroyer AL	2013	United States	Describe the process of preparing the school for a new visit by the Liaison Committee on Medical Education following a previous visit that generated a graded report	Observational and descriptive—case report	Case report	Changes in school management and culture, changes in preparation generated permanent changes. Creation of multidisciplinary work groups, balance between short‐ and long‐term objectives, reinforced a sense of urgency and purpose, clarified roles, responsibilities and deadlines, in addition to establishing leaders and stakeholders.	Curriculum and governance changes School recognition/Student satisfaction
6	Cueto J, Burch VC, Adnan NAM, et al.	2006	United States	Identify similarities and differences in accreditation practices in nine developing countries (Argentina, India, Kenya, Malaysia, Mongolia, Nigeria, Pakistan, Philippines, and South Africa) and compare them with the United States.	Observational and descriptive—case series	Qualitative data analysis	Consequences of accreditation: guarantees the quality of programmes according to defined and reliable standards; prestige and honour for the institution; attractiveness of the school for future students and families; national and international recognition of diplomas; access to grants and financing; CQI culture; identification of areas for planning and improvement; ranking of schools (competitiveness that leads to the search for quality); recognition by peers (especially important in countries where there is an increase in the number of medical schools).	CQI Curriculum and governance changes School recognition/Student satisfaction
7	Domingo F, Martinez V.	2017	Mexico	To determine whether medical education programmes recognized for their quality present better results in the National Exam for Candidates for Medical Residency	Observational and analytical—case series	Analysis of results collected from accreditation agencies	Positive correlation between the quality of the accreditation system and student exam results.	Student performance
8	Gibson KA, Boyle P, Black DA, Cunningham M, Grimm MC, McNeil HP	2008	Australia	Describe the reorganization process following feedback from the accreditation agency	Observational and descriptive—case report	Case report	The educational programme evaluation system has been revised. This system evaluates the quality of the programme based on four main aspects: curriculum and resources, teaching staff and professors, student experience, and student and alumni outcomes.	Curriculum and governance changes
9	Hedrick JS, Cottrell S, Stark D, et al.	2019	United States	Describe how 10 medical schools implemented CQI following the new LCME guidelines	Observational and descriptive—research	Remote research	Implementation is slow, and there is often more interest in the elements and criteria (theory to meet the LCME) than in actually transforming practice through CQI. Two schools stated that they would like closer guidance from the LCME throughout the implementation and indicator analysis process. All schools would like to participate in monthly or biweekly meetings with the LCME, if offered.	CQI
10	Kassebaum DG, Cutler ER, Eaglen RH.	1997	United States	To examine educational changes or reforms resulting from the accreditation process	Observational descriptive—case series	Review of survey database and site visit reports from 90 schools that have had comprehensive LCME accreditation surveys	Significant deficiencies were found in the educational programmes of 61 of the 90 medical schools that underwent accreditation surveys between 1992 and 1997, and when these 61 schools were subsequently inspected, 34 had undertaken comprehensive reforms to correct the problems or were about to do so.	Curriculum and governance changes
11	Sebiany AM, Jones Nazar CC, Mohapatra S.	2016	Saudi Arabia	To assess student perception and satisfaction with the quality of the integrated curriculum at the School of Medicine, which was changed so that the school could obtain accreditation in the previous year	Observational descriptive—research	Remote survey	Student satisfaction with the quality of the curriculum. Pointed out the need for CQI to maintain the changes implemented and the positive evaluation.	School recognition/Student satisfaction CQI
12	Shroyer AL, Lu WH, Chandran L.	2016	United States	Describe the reorganization process following feedback from the accreditation agency	Observational and descriptive—case report	Case report	Improved formative and summative feedback, enabled curriculum improvement, increased stakeholder engagement, mapped key actors outside the school who began to participate and take responsibility for the curriculum process, expanded teacher training, addressed disrespect for mapped students – implemented training to address these issues	Curriculum and governance changes
13	van Zanten M, Mckinley D, Durante Montiel I, Pijano C V.	2012	Mexico and Philippines	To investigate the potential impact of medical school accreditation on student performance on certification examinations	Observational analysis—cohort	Cohort of Mexican and Philippine citizens who attended medical schools	Positive correlation between school accreditation and student exam results.	Student performance
14	van Zanten M.	2015	United States	Searching for an association between the quality of the accreditation system of the student's home country and his/her performance on the validation exams for entry into the United States	Observational analysis—cross‐sectional cohort	Remote research with data analysis	Positive correlation between the quality of the accreditation system and student exam results.	Student performance
15	Akdemir N, Peterson LN, Campbell CM, Scheele F.	2020	Netherlands	Evaluates the pros and cons of CQI in educational institutions that have traditionally been accredited based on episodic assessments by external reviewers.	Qualitative	Seven criteria relevant to government oversight were used to assess the pros and cons of using CQI in three medical school accreditation systems across the medical education continuum.	Because institutional CQI makes use of early warning systems, it can enhance the reflective function of accreditation. CQI, as assessed using the seven oversight criteria, has pros and cons. A toxic culture that undermines impartiality and independence, as well as the need to invest in bureaucratic systems, may make it impractical for some institutions to conduct CQI.	CQI
16	Al‐ Eyadhy A, Alenezi S.	2021	Saudi Arabia	To assess the extent to which external quality assurance practices have prevented accreditation processes from being reflected at the other end of the learning pathway, including student satisfaction.	Quantitative retrospective analysis of secondary data	A before‐and‐after comparison research design was based on a retrospective analysis of quantitative secondary data and the outcome was measured by calculating the average annual student satisfaction score of all eligible courses for each academic year and analysing their changes over time before and after the accreditation processes.	Both accreditation cycles were associated with higher student satisfaction scores. Preparatory phase activities and navigating the self‐study assessment while challenging programme competencies are essential triggers for quality improvement practices associated with accreditation.	School recognition/Student satisfaction
17	Al‐ Shehri AM, Al‐Alwan I.	2013	Saudi Arabia	It describes the development of accreditation in Saudi Arabia and suggests a strategy for creating a culture of quality in medical schools in preparation for meaningful accreditation systems that ensure adequate participation of all stakeholders in evidence‐based quality management.	Descriptive	Not applicable	It describes the development of accreditation in Saudi Arabia and suggests a strategy for creating a culture of quality in medical schools in preparation for meaningful accreditation systems that ensure adequate participation of all stakeholders in evidence‐based quality management.	Curriculum and governance changes
18	Alenezi S, Al‐ Eadhy A, Barasain R, AlWakeel TS, et al.	2023	Saudi Arabia	Evaluate the impact of external accreditation on student GPAs during an accreditation cycle.	A quantitative retrospective analysis of secondary data	A before‐and‐after comparison research project to assess the impact of external accreditation on student GPAs during an accreditation cycle	Pre‐ and post‐accreditation revealed a statistically significant improvement in students' average grades. The planning phase and the self‐assessment journey not only verified the programme's competencies but also acted as critical drivers for quality improvement processes and therefore for students' learning experiences.	Student performance Curriculum and governance changes
19	Amaral E, Norcini J.	2022	Brazil	Provide an overview of key quality assurance, accreditation, and licencing strategies in health professions education.	Descriptive	Not applicable	Clear evidence of effectiveness, especially for accreditation, is scarce and difficult to obtain, particularly when it comes to health outcomes.	Curriculum and governance changes
20	Arja SB, Arja SB, Fatteh S.	2021	Canada	Investigating Caribbean medical school leaders' perceptions of the impact of accreditation on their school's processes.	Qualitative study and data analysis	A qualitative study and data analysis were done using framework analysis.	Themes of processes influenced by accreditation in Caribbean medical schools were similar to those found in the Canadian context and align with CQI best practices. Caribbean medical schools are changing their educational processes as a result of accreditation requirements.	CQI Curriculum and governance changes
21	Benbassat J, Baumal R, Cohen R.	2022	Israel	Suggest that medical schools be involved in ongoing monitoring of their teaching programmes for compliance with accreditation standards.	Descriptive	Not applicable	Medical education units are tasked with overseeing accreditation readiness through ongoing self‐assessment/monitoring of the implementation of medical school education programmes. In fact, such ongoing monitoring has been demonstrated at 10 US medical schools to improve the learning environment, career guidance, teaching physical examination, internship feedback, and communication with faculty and other stakeholders.	CQI
22	Blouin B, Tekian A, Kamin C, et al.	2018	Canada	Assessing the impact of accreditation using an innovative marker: the processes implemented in medical schools as a result of accreditation.	Qualitative study	Interviews and focus group discussions with deans, directors of undergraduate medical education, and faculty leaders from 13 of 17 Canadian medical schools were used to obtain perspectives on processes influenced by accreditation; the constant comparative analysis method associated with grounded theory was used to generate process themes.	Establishment of processes related to governance, academic accountability, data collection and analysis, monitoring, documentation, and policy creation and review. Accreditation also contributes to CQI and faculty engagement in the medical education programme. Counterbalancing the impact on school processes, several negative consequences of accreditation for medical education programmes have been identified.	Curriculum and governance changes CQI
23	Choa G, Arfeen Z, Chanc SCC, et al.	2022	United Kingdom	Systematically identified and synthesized qualitative studies that explored the accreditation experiences of medical faculty and students.	Descriptive	Literature review ‐ Four databases (PubMed, EMBASE, ERIC and PsychINFO) were searched for relevant published articles.	Divergent views on the value of medical school accreditation from students and staff, including both positive and negative impacts on medical education programmes and stakeholders. Consequences include those on staff morale, student‐faculty relationships, and faculty workloads. Medical faculty also have a number of concerns about the credibility of accreditation standards, evaluators, and processes. Regulators and policymakers should consider the views of faculty and students as they seek to improve current accreditation practices.	Curriculum and governance changes Negative points
24	Jung H, Jeon WT, An S	2020	Korea	Help medical schools make the most of the accreditation process as an opportunity for educational development.	Descriptive	‘First, the meaning and objectives of accreditation are explained. Second, we introduce accreditation standards and elements and present a comparison between Korea's model of the accreditation process and the models of other countries. Third, we discuss how the pedagogical value of education has been realized in the field of medical education. Finally, based on these discussions, we propose directions for the accreditation body and medical schools to improve the quality of medical education’.	Propose collaboration between medical schools, academic societies and the Korea Association of Medical Colleges with the consent of the accrediting body to provide an opportunity for formative assessment, although this is not yet concrete. Through these steps, medical schools across the country can jointly develop and share a new culture in which accreditation is a real opportunity for development and change.	Proposal for sharing accreditation data
25	Javidan AP, Raveendran L, Rai Y, et al.	2020	Canada	Propose that increasing transparency in credentialing could increase trust in the institutions that produce society's doctors.	Descriptive	Search of the websites of each of the 17 medical schools in Canada (including a manual search, as well as the search terms ‘accreditation’, ‘independent student review’ and ‘self‐study’, as well as their combinations and variants in French)	The effects of transparency of these accreditation findings on public trust have not been studied. However, it seems reasonable to infer that public reporting of accreditation results would allow stakeholders to view accreditation data that may have previously been inaccessible. In turn, they can assess whether medical schools are operating within their interests, which may provide public confidence in an effective accreditation process.	Proposal for sharing accreditation data
26	Nora LM	2022	United States	A review by the author of this commentary of such consultations across 17 schools showed variability in how diversity, equity, and inclusion (DEI) information was incorporated and discussed in accreditation‐related materials and interviews.	Descriptive	Over a 30‐month period of work with 17 LCME‐accredited medical education programmes, which included review of accreditation‐related documents, stakeholder interviews, midcycle gap analyses, and simulated site visits, the teams in this case found that all schools made diligent efforts to promote diversity, equity, and inclusion (DEI).	Although few LCME accreditation standards explicitly address diversity, equity and inclusion (DEI), it is an important component of the educational programme, the hidden curriculum and the lived experience of students and faculty. Schools that examine their DEI efforts across the full range of LCME standards may see opportunities to strengthen their programmes, highlight their successes, or both.	Curriculum and governance changes
27	Paredes, E.	2008	Peru	Define and discuss aspects related to the medical accreditation process in Latin America	Descriptive	Not applicable	Accreditations provide the institution with an opportunity for self‐assessment and ‘knowing itself’ and establishing what its weaknesses and strengths are, planning and developing improvement plans and integrating its activities.	Curriculum and governance changes
28	Rosselot EJ	2001	Chile	Present and discuss the accreditation process of medical schools in Chile	Descriptive	Not applicable	It is clear that self‐assessment constitutes an effective tool for broad and comprehensive analysis, very useful for providing up‐to‐date knowledge of the institution, its strengths and weaknesses, its capacity and potential, its limitations and how to overcome them. Although carried out by stakeholders involved in the process being assessed, in terms of their institutional affiliation, it is possible to carry out the different stages of accreditation with sufficient impartiality. In fact, the process can be characterized by excessive self‐criticism rather than acceptance of the status quo, as stakeholders have been able to confirm the virtues and objectivity of the accreditation process by contrasting its conclusions with those of external peers and subsequent accreditation.	Curriculum and governance changes
29	Roy M, Wood TJ, Blouin D, et al.	2020	Canada	To investigate the relationship between the accreditation cycle and performance on a national licencing examination.	Descriptive	Scores on the computer‐based portion of the Medical Council of Canada Qualifying Examination, Part I, from 1993 to 2017 were examined for all 17 Canadian medical schools. Typically completed after graduation from medical school, results within each year were transformed for comparability across administrations and linked to time within each school's accreditation cycle. ANOVAs were used to assess the relationship between time since accreditation and examination scores. Secondary analyses isolated 4‐year versus 3‐year training programmes and separated data generated before versus after the implementation of a national informal midcycle review programme.	Formal, externally conducted accreditation cycles appear to be associated with educational processes in ways that have translated into improved student outcomes on a national licensure examination.	Student performance
30	van Zanten M, Boulet JR, Shifer CD	2022	Canada	To compare the performance on the United States Medical Licensing Examination (USMLE®) of students and graduates who attended international medical schools accredited by an agency recognized by the World Federation for Medical Education with individuals who attended schools that did not meet this criterion.	Cohort	Cross‐performance on the USMLE (pass/fail result on the first attempt) and medical school accreditation status.	Individuals seeking certification from the Educational Commission for Foreign Medical Graduates who attended international medical schools accredited by an agency recognized by the World Federation of Medical Education had higher or comparable first‐attempt pass rates on the United States Medical Licensing Examination compared to individuals who attended medical schools that did not meet this criterion. Important positive evidence that external evaluation of educational programmes is associated, on average, with better educational outcomes.	Student performance
31	You Y, Li M, Xie A, et al.	2023	China	Illustrate how the establishment and initial implementation of an accreditation system influences medical school performance on the national medical licencing examination.	Retrospective observational study	Data analysis of 105 Chinese medical schools during accreditation (2012–2021) and medical licencing examination pass rates (2011–2019), as compared with 834 academic year records in a window of years before and after accreditation. The authors employed fixed‐effects regression models with a comparison group to exclude factors that may have confounded the impacts of accreditation timing. They also demonstrated the heterogeneous effects of accreditation by level and achievement gap of medical schools.	Substantial cumulative improvement (more than 15 percentage points) in pass rates during the years prior to accreditation, with no clear trend indicating performance declines in the years after accreditation. Lower‐tier medical schools saw greater benefits from accreditation. Medical schools with a larger prior performance gap achieved a larger percentage point increase in pass rates over time in the preaccreditation years.	Student performance

Abbreviations: CQI, continuous quality improvement; LCME, Liaison Committee for Medical Education.

We included 31 studies carried out in the context of undergraduate medical courses and which reported some type of perceived impact on the medical school after going through the accreditation process. These studies were published between 1997 and 2022 in the following countries: United States (*n* = 8), Canada (*n* = 7), Saudi Arabia (*n* = 5), Mexico and Philippines (*n* = 2), Australia (*n* = 1), Brazil (*n* = 1), Chile (*n* = 1), China (*n* = 1), Israel (*n* = 1), Korea (*n* = 1), Netherlands (*n* = 1), Peru (*n* = 1) and United Kingdom (*n* = 1).

In terms of the methodology used to investigate the impacts of the accreditation process, the studies applied focus groups and interviews [16, 17, 18], case series [19, 20, 21, 22, 23, 24, 25, 26, 27, 28, 29], case reports [30, 31, 32, 33, 34, 35, 36, 37, 38], remote surveys [39, 40, 41], cohort study [42] and literature reviews [43, 44]

Heterogeneity was identified between the accreditation processes reported in the studies. The main differences are related to the evaluation criteria used by accreditation agencies. Furthermore, there are differences between the agencies themselves, such as the fact that it is not exclusive to medical courses in some countries.

For a better analysis of the impacts of accreditation described, we grouped the studies, according to the results, into six categories, regarding the impacts of accreditation: curriculum and governance changes, Continuous Quality Improvement (CQI), student performance, school recognition/student satisfaction, negative points and proposal for sharing accreditation data.

Curriculum and governance changes were cited by 16 studies [17, 18, 19, 20, 23, 25, 26, 30, 32, 33, 34, 35, 43, 44, 45, 46], and CQI appeared as an impact of accreditation in 11 studies [16, 17, 18, 20, 23, 29, 31, 36, 39, 40, 45]. Of the studies reviewed, eight cited improved exam performance by students graduating from accredited medical schools [18, 21, 27, 28, 35, 38, 41, 47]. School recognition and student satisfaction were impacts cited in five of the studies included in this review [18, 20, 22, 32, 40]. A proposal to create a system for sharing data on accreditation processes was presented by two studies [24, 37].

On the other hand, three studies cited negative aspects of accreditation, such as stress, financial demands and dissatisfaction with the current accreditation process [16, 43, 48].

Of the studies selected for inclusion in this review, two of them [32, 40] provided reports and data on impacts of accreditation directly related to the feedback obtained in the evaluation process.

## 4 Discussion

This review aimed to identify the impacts of the accreditation process on medical schools, specifically undergraduate courses. Studies that address the impacts of medical school accreditation are relatively scarce given the magnitude of the topic of medical education.

The 31 included studies reported positive and negative aspects related to the accreditation process as well as the challenges associated with the process per se. The results showed an increase in the number of publications that addressed the impacts of accreditation on medical schools in recent years. Improvements in students' performance during exams were reported as positive consequences of accreditation, whereas the associated stress of being evaluated, the high costs and the lack of transparency were negative aspects reported by the stakeholders involved in the process.

Only three studies included in this scoping review pointed out negative aspects as an outcome of the accreditation process for medical schools. These negative aspects included stress, temporary changes to comply with the checklist of the accreditation process [16] demand for financial and human resources that leads to loss of investment opportunities in other areas, potential impact on faculty and staff morale, and internal conflicts among team members resulting from the accreditation process [43]. Other negative aspects mentioned concern the organization of the accrediting agencies themselves and were not considered impacts on the schools [45].

Monitoring for realistic transformation and not just temporarily meeting established criteria could minimize the risk of ‘accreditation burnout’, which, in turn, leads only the negative points of such an important process to be emphasized [28].

Two studies [32, 40] indicated the impact of accreditation directly related to the feedback obtained in the evaluation process. If it is not well structured with well‐defined impact markers, accreditation may represent a false assessment of quality now of the test only, not reflecting on the quality of care or education provided [49]. Therefore, what is expected is ‘sustainable accreditation’, with effective transformations in schools that lead to lasting results [41].

According to the categorization of the results of the studies included in this review, CQI was cited as a significant impact of accreditation for medical schools in terms of maintaining the process of continuous improvement and not just at the time of the evaluation. Thus, CQI is recognized as a positive impact of accreditation in medical schools. CQI must promote a change in organizational culture in schools so that the changes last beyond the requirements of the accrediting agencies and produce real improvements [16, 17, 18, 20, 23, 29, 31, 36, 39, 40, 45].

Still observing the categorization of the results, the curriculum and governance changes occurred frequently in the accredited medical schools, having been mentioned as positive changes, such as an increase in the sense of responsibility, engagement and belonging to the team, review of internal policies [48] creation of research [18] and multidisciplinary groups [46] elucidation of the roles of the actors involved and the purpose of the institution [46]. Most of the results included in this category concern permanent changes in the school, which are linked to the change in culture and, consequently, refer to CQI.

Accreditation is meaningful only when there is mutual trust between evaluators and the evaluated medical schools, establishing an ‘educational alliance’

If schools see accreditation as an opportunity to implement innovations or a tool to reorganize the curriculum, real transformations can occur, contributing to political decision‐making, serving as a catalyst for medical education reform, promoting education aimed at continuous learning and protecting society from the consequences of insufficient training [37].

Another category of results concerns the recognition and satisfaction of the school by stakeholders, such as prestige and honour for the institution; attractiveness of the school for future students and families; national and international recognition of diplomas; and access to grants and financing [18, 20] mentions that, sometimes, these interferences are perceived indirectly in relation to accreditation.

Studies have reported a positive correlation between school accreditation and the performance of graduates in medical practice exams in the United States [21, 24, 38]. By relating performance on such tests and the quality of accreditation agencies in candidate countries, van Zanten [24, 27, 41] confirmed the importance of a high‐quality accreditation system that assesses skills in real‐life scenarios.

Accrediting agencies may be specifically assigned to the accreditation of medical schools or, in general, to undergraduate institutions. These agencies can also be linked to government bodies or even have mixed councils. There are countries where accreditation is mandatory for all practising medical schools and others where it is optional. The cost of accreditation for schools varies widely, from no cost in some countries to a high cost in others.

If the studies had provided the results of the diagnostic evaluation, it would be possible to identify common weak points and the strategies adopted to address them. A more detailed final report of accreditation process could provide information about the improvements made and perceptions of the effects of implementing the changes.

In addition, there are no validated and standardized instruments for evaluating the effects of accreditation in medical schools to allow the impacts to be measurable through the analysis of previously established criteria.

Considering all the above‐mentioned differences, the standardization of an instrument for measuring the impacts of the accreditation process seems to be a complex option; however, accrediting agencies might consider monitoring the outcomes of the evaluation processes in the daily life of schools and their sustainability throughout the duration of the validity of the accreditation.

In this sense, the proposal to create a system or process for sharing data on accreditation processes, which was cited as a result by two studies included in this review [24, 37], seems to be an interesting option for improving accreditation systems around the world.

The process of medical school accreditation needs to be increasingly improved, becoming part of the agenda of priority processes for ensuring the quality of medical education

[50]. We believe that, to the extent that it serves as evidence of quality, accreditation can drive improvements in the content, format and perspective of educational practice and, even more so, can be used strategically to foster research and development in medical education [3].

It is recommended that the evaluation takes place in a systematic way and with constant dialogue between evaluators so that the process is not considered punitive and the presence of elements of surprise is avoided. The process must be conducted with a focus on the definitive transformation of reality.

### 4.1 Study Limitations

The electronic search was carried out using terms in English, and no restriction filters were applied to the language or year of publication. However, no searches were carried out in other languages or specific databases from European, Asian and African countries, which probably limited the identification of studies about the accreditation process in these countries.

Most of the included studies did not report the results of accreditation considering the direct feedback obtained through the evaluation process. This lack of transparency about the accreditation criteria hindered an analysis of these criteria and a critical discussion of the results obtained and criteria evaluated during the accreditation process.

The lack of prior registration of the protocol for this review must also be cited as a limitation of the study.

## 5 Conclusion

The accreditation process has positive effects, such as promoting institutional changes, self‐evaluation, improving the performance of graduates in certification exams, increasing trust from stakeholders and increasing the satisfaction of professionals and students, in addition to improving organizational culture and CQI culture.

At the same time, negative points were also mentioned, such as the need for large investments of temporal, financial and human resources that contribute to heightened stress for the school, which can lead to resistance from schools to the accreditation process.

We strongly recommend that the accreditation experiences of medical schools in different countries be shared and reported in greater detail, especially in relation to criteria/guidelines. With clear communication of this information, the outcomes of interest may be properly selected and evaluated through valid and reliable instruments.

As future recommendations, it would be pertinent to invest in studies that demonstrate the impacts of the accreditation process for medical schools in terms of social responsibility, as well as responsibility towards regional health systems.

Generating evidence on the impacts of accreditation for medical schools should be understood as a mechanism for expanding this emerging area of study for medical education

In addition, related studies could increase the visibility of the need for minimum quality standards in the training of physicians and broaden the discussion on the topic around the world.

## Author Contributions

**Leticia C. Girotto:** investigation, writing – original draft, conceptualization, data curation, supervision, project administration, formal analysis, validation, writing – review and editing. **Karynne B. Machado:** investigation, validation, formal analysis, data curation. **Roberta F. C. Moreira:** methodology, validation, writing – review and editing, writing – original draft. **Milton A. Martins:** writing – review and editing, validation, writing – original draft. **Patrícia Z. Tempski:** validation, formal analysis, supervision, writing – original draft, conceptualization.

## Ethics Statement

The authors have nothing to report.

## Consent

The authors have nothing to report.

## Conflicts of Interest

The authors declare no conflicts of interest.

## Data Availability

All data generated or analysed during this study are included in this published article (Table 1). The other information (papers) is in the public domain and can be accessed in the respective databases and journals. Any other datasets used and/or analysed during the current study are available from the corresponding author upon reasonable request.

## Appendix A

**TABLE A1:** Strategies of electronic and structured searches.

Databases or repositories		
Source	Search strategy	Results
Eric	#1 Medical Schools OR Medical School OR School #2 Accreditation OR accreditation process #3 Improvement, Quality OR Improvements, Quality OR Quality Improvements #1 AND #2 AND #3	1.926
PubMed	#1 MeSH descriptor: [accreditation] explode all trees #2 MeSH descriptor: [medical school] explode all trees #3 MeSH descriptor: [quality improvement] explode all trees #4 ‘Medical School’ OR ‘School, Medical’ OR ‘Medical Schools’ #5 ‘Improvement’ OR ‘Quality OR Improvements’ OR ‘Quality’ OR ‘Quality Improvements’ OR ‘QI’ #1 AND #2 AND #3 explode all trees #6 #1 AND #4 AND #5	577
EMBASE (via Elsevier)	#1 ‘Accreditation’/exp #2 ‘Medical school’/exp #3 ‘Quality improvement’/exp #4 ‘Medical School’ OR ‘School, Medical’ OR ‘Medical Schools’ #5 ‘Improvement’ OR ‘Quality OR Improvements’ OR ‘Quality’ OR ‘Quality Improvements’ #1 AND #2 AND #3 #6 #1 AND #4 AND #5	1198
Web of Science	#1 ‘Accreditation’ #2 ‘Medical School’ OR ‘School, Medical’ OR ‘Medical Schools’ #3 ‘Improvement’ OR ‘Quality OR Improvements’ OR ‘Quality’ OR ‘Quality Improvements’ #1 AND #2 AND #3	808
Lilacs (via BVS)	#1 Accreditation OR ‘accreditation process’ #2 ‘Medical School’ OR ‘Medical Schools’ #3 ‘Quality Improvements’ #4 #1 AND #2 #4 #1 AND #2 AND #3	40
CINAHL (via BVS)	#1 Accreditation OR ‘accreditation process’ #2 ‘Medical School’ OR ‘Medical Schools’ #3 ‘Quality Improvements’ #4 #1 AND #2 #4 #1 AND #2 AND #3	203

Grey literature		
Source	Search strategy	Results
Opengrey	(‘Accreditation’ OR ‘accreditation process’) AND (‘Medical School’ OR ‘Quality Improvements’)	1
Open Access Theses and Dissertations	‘Accreditation’ AND ‘Medical School’ AND ‘Quality improvements’	0

Abbreviations: BVS, Biblioteca Virtual em Saúde; LILACS, Literatura Latinoamericana y del Caribe en Ciencias de la Salud; MEDLINE, Medical Literature Analysis and Retrieval System Online; PMC, PubMed Central.
